# CRISPR-mediated generation and characterization of a *Gaa* homozygous c.1935C>A (p.D645E) Pompe disease knock-in mouse model recapitulating human infantile onset-Pompe disease

**DOI:** 10.1038/s41598-022-25914-8

**Published:** 2022-12-14

**Authors:** Shih-hsin Kan, Jeffrey Y. Huang, Jerry Harb, Allisandra Rha, Nancy D. Dalton, Chloe Christensen, Yunghang Chan, Jeremy Davis-Turak, Jonathan Neumann, Raymond Y. Wang

**Affiliations:** 1 CHOC Children’s Research Institute, Orange, CA 92868 USA; 2 School of Medicine, New York Medical College, Valhalla, NY 10595 USA; 3 ROSALIND, San Diego, CA 92126 USA; 4 Transgenic Mouse Facility, University of California Irvine, Irvine, CA 92697 USA; 5 Division of Metabolic Disorders, CHOC Children’s Specialists, Orange, CA 92868 USA; 6 Department of Pediatrics, University of California-Irvine, Irvine, CA 92697 USA

**Keywords:** Genetics, Mutation, Metabolic disorders, Molecular medicine, Genetics research, Genetic engineering, Gene therapy, Targeted gene repair

## Abstract

Pompe disease, an autosomal recessive disorder caused by deficient lysosomal acid α-glucosidase (GAA), is characterized by accumulation of intra-lysosomal glycogen in skeletal and oftentimes cardiac muscle. The c.1935C>A (p.Asp645Glu) variant, the most frequent *GAA* pathogenic mutation in people of Southern Han Chinese ancestry, causes infantile-onset Pompe disease (IOPD), presenting neonatally with severe hypertrophic cardiomyopathy, profound muscle hypotonia, respiratory failure, and infantile mortality. We applied CRISPR-Cas9 homology-directed repair (HDR) using a novel dual sgRNA approach flanking the target site to generate a *Gaa*^*em1935C*>*A*^ knock-in mouse model and a myoblast cell line carrying the *Gaa* c.1935C>A mutation. Herein we describe the molecular, biochemical, histological, physiological, and behavioral characterization of 3-month-old homozygous *Gaa*^*em1935C*>*A*^ mice. Homozygous *Gaa*^*em1935C*>*A*^ knock-in mice exhibited normal *Gaa* mRNA expression levels relative to wild-type mice, had near-abolished GAA enzymatic activity, markedly increased tissue glycogen storage, and concomitantly impaired autophagy. Three-month-old mice demonstrated skeletal muscle weakness and hypertrophic cardiomyopathy but no premature mortality. The *Gaa*^*em1935C*>*A*^ knock-in mouse model recapitulates multiple salient aspects of human IOPD caused by the *GAA* c.1935C>A pathogenic variant. It is an ideal model to assess innovative therapies to treat IOPD, including personalized therapeutic strategies that correct pathogenic variants, restore GAA activity and produce functional phenotypes.

## Introduction

Glycogen storage disease type II, also called Pompe disease (PD; OMIM#232300), is an autosomal recessive disorder resulting from malfunction of lysosomal acid α-glucosidase (GAA; EC 3.2.10.20) caused by mutations in the *GAA* gene (OMIM#606800). GAA deficiency leads to reduced glycogen degradation and accumulation of intra-lysosomal glycogen with pronounced glycogen storage in cardiac and skeletal muscle. Increased glycogen storage in myocytes, brain, and spinal cord anterior horn neurons results in muscle weakness, which varies in age of onset and severity according to the level of residual GAA enzymatic activity^1^. PD presents as a spectrum of phenotypes, typically classified into infantile-onset form (IOPD) and late-onset form (LOPD) based on the time of disease onset^2–4^. Patients with severe IOPD have neonatal onset and a rapidly progressive disease with prominent cardiomyopathy, general muscle weakness and hypotonia, respiratory problems and drastically reduced life expectancy. Patients with LOPD have a more slowly progressive proximal skeletal myopathy eventually resulting in mobility problems and respiratory difficulties, but generally do not present with hypertrophic cardiomyopathy^3^.

Recombinant GAA (rhGAA) enzyme replacement therapy (ERT) was developed to treat PD and approved by the FDA in 2006. ERT improves the survival of patients and is very effective at reducing glycogen levels in heart muscle and reversing cardiac symptoms. However, only partial recovery of muscle strength can be achieved with ERT. Surviving children still have glycogen buildup in other muscles and experience challenges performing basic activities such as walking, speech enunciation, eating or even breathing^5,6^.

The *GAA* gene has a very heterogeneous mutational spectrum, with more than 900 *GAA* variants documented in the Pompe disease GAA variant database^7–9^. Among these variants, the *GAA* c.1935C>A transversion in exon 14, which results in the p.Asp645Glu (p.D645E) missense mutation, is the most frequent pathogenic variant associated with IOPD in the Southern Chinese, Taiwanese, and Southeast Asian populations of Han ancestry, but is not frequently reported in any other region^8,10^. In Taiwanese populations, this c.1935C>A variant represents 36–80% of mutations^11,12^, and occur in context of a specific haplotype with conserved polymorphic markers linked to Taiwanese Pompe IOPD patients comparing to normal individuals. This may suggest the existence of a founder effect stemming from a diaspora of Southern Han Chinese to Taiwan and other locations^12^.

Here, we report the generation of a *Gaa*^*em1935C*>*A*^ (p.D645E) knock-in (KI) mouse model of PD by CRISPR-Cas9 homology-directed repair (HDR) using a dual sgRNA approach. The primary objective of this study is to characterize the molecular, biochemical, physiological, histological, and behavioral phenotypes of this KI mouse model. We anticipate that this novel *Gaa*^*em1935C*>*A*^ mouse model will be a valuable research tool, especially when compared to other *Gaa* knock-out (KO) and KI models. Altogether, preclinical KI models of PD will further accelerate our understanding of how pathogenic *GAA* mutations result in variable disease onset, progression, and response to current and future therapeutic strategies.

## Results

### ***Gaa***^***c.1935***^ target locus guide RNA and donor ssODN design

In silico design of CRISPR-Cas9 guide RNAs (gRNAs) specific for the *Gaa*^*c.1935*^ target locus was initially performed using CRISPick, the Genetic Perturbation Platform (GPP) sgRNA Designer^13^. Candidate gRNAs were selected using the following criteria: 1) top combined rank score (based on on-target efficacy and off-target specificity scores) and 2) proximity of predicted Cas9 nuclease cut site to the *Gaa*^*c.1935*^ target locus. Further potential gRNA off-target analysis was performed using Genome Target Scan (GT-Scan)^14^. Two gRNAs were first selected to be used in generating *Gaa*^*c.1935C*>*A*^ KI C2C12 cells: gRNA-1 (5′- CGCAGATGTCCGCCCCGACC-3′), and gRNA-2 (5′- GCAGATGTCCGCCCCGACCA-3′).

### Generation and characterization of *Gaa*^*c.1935C*>*A*^ KI C2C12 cell line

**Figure 1:**
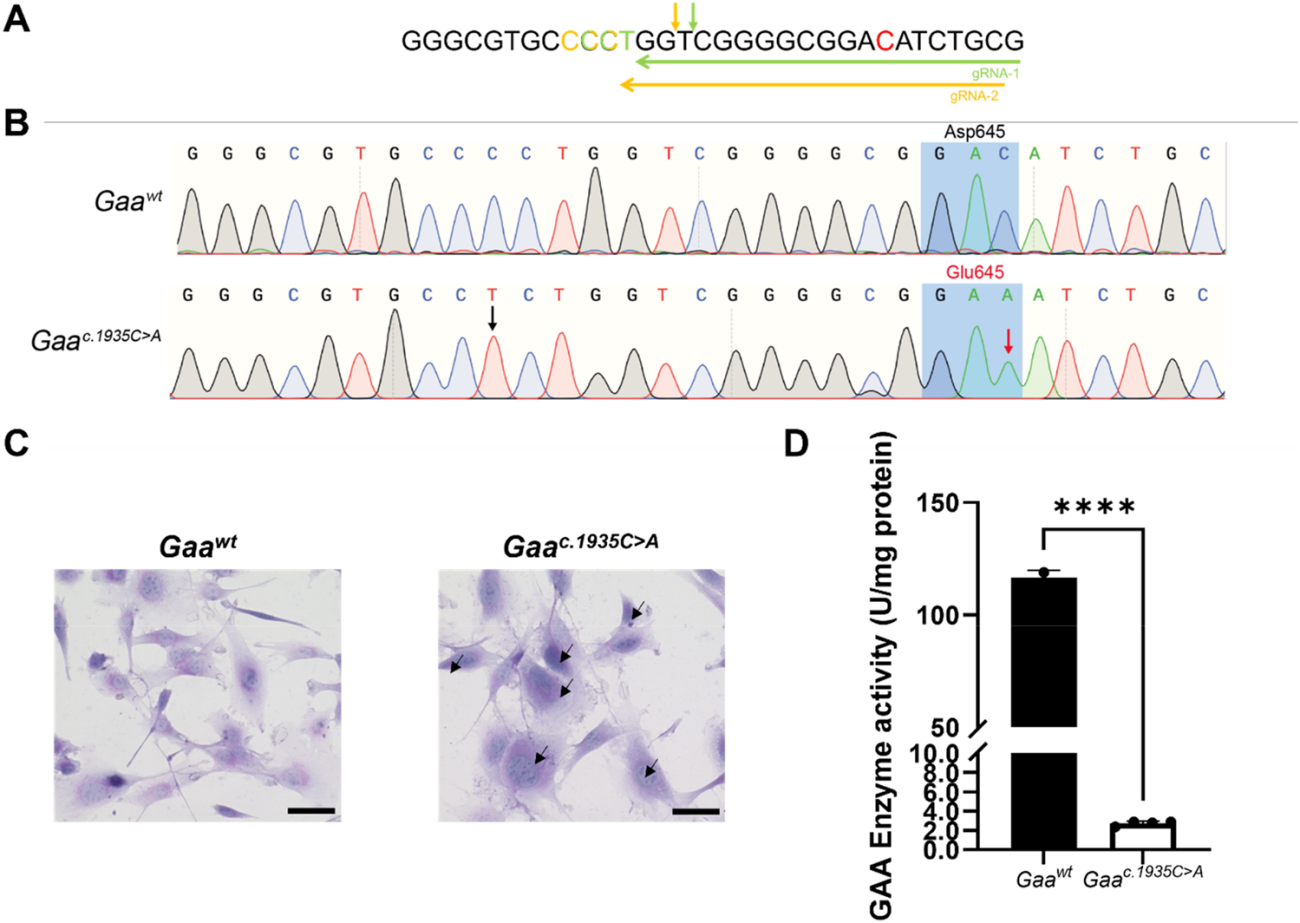
Generation and characterization of a *Gaa*^*c.1935C*>*A*^ C2C12 myoblast clonal cell line. (**A**) Sequences of guide RNAs targeting the *Gaa*^*c.1935*^ target locus. Horizontal arrow indicates antisense guide RNAs used in this study. Protospacer adjacent motifs (PAM; NGG) are highlighted in color corresponding to the respective guide RNA. The *Gaa*^*c.1935*^ locus for targeted cytosine to adenine transversion is highlighted in red. (**B**) Sanger sequencing chromatograms of controls (*Gaa*^*wt*^) and clonal KI (*Gaa*^*c.1935C*>*A*^) C2C12 myoblast genomic DNA at the *Gaa*^*c.1935*^ locus. Black arrow indicates a synonymous mutation at the PAM site (*Gaa*^*c.1920C*>*T*^). Red arrow indicates the desired KI mutation (*Gaa*^*c.1935C*>*A*^). Gray shaded region indicates amino acid change from aspartic acid (Asp; GAC) to glutamic acid (Glu; GAA) at position 645. (**C**) Periodic-acid Schiff (PAS) staining of control (*Gaa*^*wt*^) and clonal KI (*Gaa*^*c.1935C*>*A*^) C2C12 myoblasts. Fixed cells were stained by PAS staining (purple-magenta) and counterstained by hematoxylin (blue). Only *Gaa*^*c.1935C*>*A*^ KI myoblasts display significant accumulated PAS staining (see arrows). Representative images were captured on a bright-field microscope at 20 × objective magnification. Scale bar represents 50 µm. (D) GAA enzymatic activity in *Gaa*^*wt*^ and *Gaa*^*c.1935C*>*A*^ C2C12 myoblasts. Very low GAA activity (~ 2.3% of wt) was measured in *Gaa*^*c.1935C*>*A*^ C2C12 myoblasts compared to *Gaa*^*wt*^ C2C12 myoblasts. GAA enzymatic activity was measured using a fluorometric 4-MU α-d-glycoside assay and normalized to total amount of sample protein. Data generated from three independent experiments are shown as mean ± SD. Comparisons were analyzed with unpaired one-tailed *t*-tests. ****p < 0.0001.

*Gaa*^*c.1935*^ gRNA-1 and gRNA-2 expression vectors and their respective single-stranded donor oligonucleotides (ssODN) were electroporated into C2C12 mouse myoblasts to assess in vitro on-target editing and HDR efficiency. *Gaa*^*c.1935*^ gRNA-2 demonstrated higher on-target editing (26.7 ± 10.7%) and HDR efficiency (5.4 ± 3.4%) than gRNA-1 (on-target editing: 13.2 ± 3.7%; HDR efficiency: 3.8 ± 0.6%) (Table 1). Following puromycin-resistant selection, we were able to successfully isolate and expand *Gaa*^*c.1935C*>*A*^ KI C2C12 clonal cells electroporated with *Gaa*^*c.1935*^ gRNA-1 and/or gRNA-2 and their respective donor ssODN (Table 1; Fig. 1A). Sanger sequence results confirmed that the *Gaa*^*c.1935C*>*A*^ KI mutation along with a *Gaa*^*c.1920C*>*T*^ silent protospacer adjacent motif (PAM) mutation were successfully introduced into the clonal line (Fig. 1B).

**Table 1:**
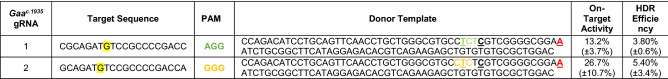
*Gaa*^*c.1935*^ guide RNA on-target activity and HDR efficiency.

The target sequence, PAM motifs and donor templates used for testing of *Gaa*^*c.1935*^ guide RNAs in C2C12 mouse mybloasts are outlined. *Gaa*^*c.1935*^ locus for each gRNA target sequence is highlighted in yellow. Desired *Gaa*^*c.1935*^ KI mutation (red), silent PAM site (either green or gold, corresponding to gRNA) and gRNA seed region (black) mutations are bolded and underlined in the donor template sequence. Total on-target Cas9 nuclease activity and HDR efficiency for each *Gaa*^*c.1935*^ guide RNA condition is displayed as the average of two independent experiments.

In comparison to *Gaa*^*wt*^ cells, *Gaa*^*c.1935C*>*A*^ KI cells displayed increased PAS staining, indicating the accumulation of glycogen (Fig. 1C). Furthermore, GAA enzymatic activity was almost abolished in *Gaa*^*c.1935C*>*A*^ KI cells relative to *Gaa*^*wt*^ cells; less than 2.3% of WT GAA activity was detected in the KI cell line (Fig. 1D). Taken together, these results demonstrate that our *Gaa*^*c.1935C*>*A*^ KI C2C12 cell line exhibits a molecular and biochemical phenotype observed in human PD and can be utilized as an in vitro model for further study.

### Generation and characterization of *Gaa*^*em1935C*>*A*^ transgenic mice

**Figure 2:**
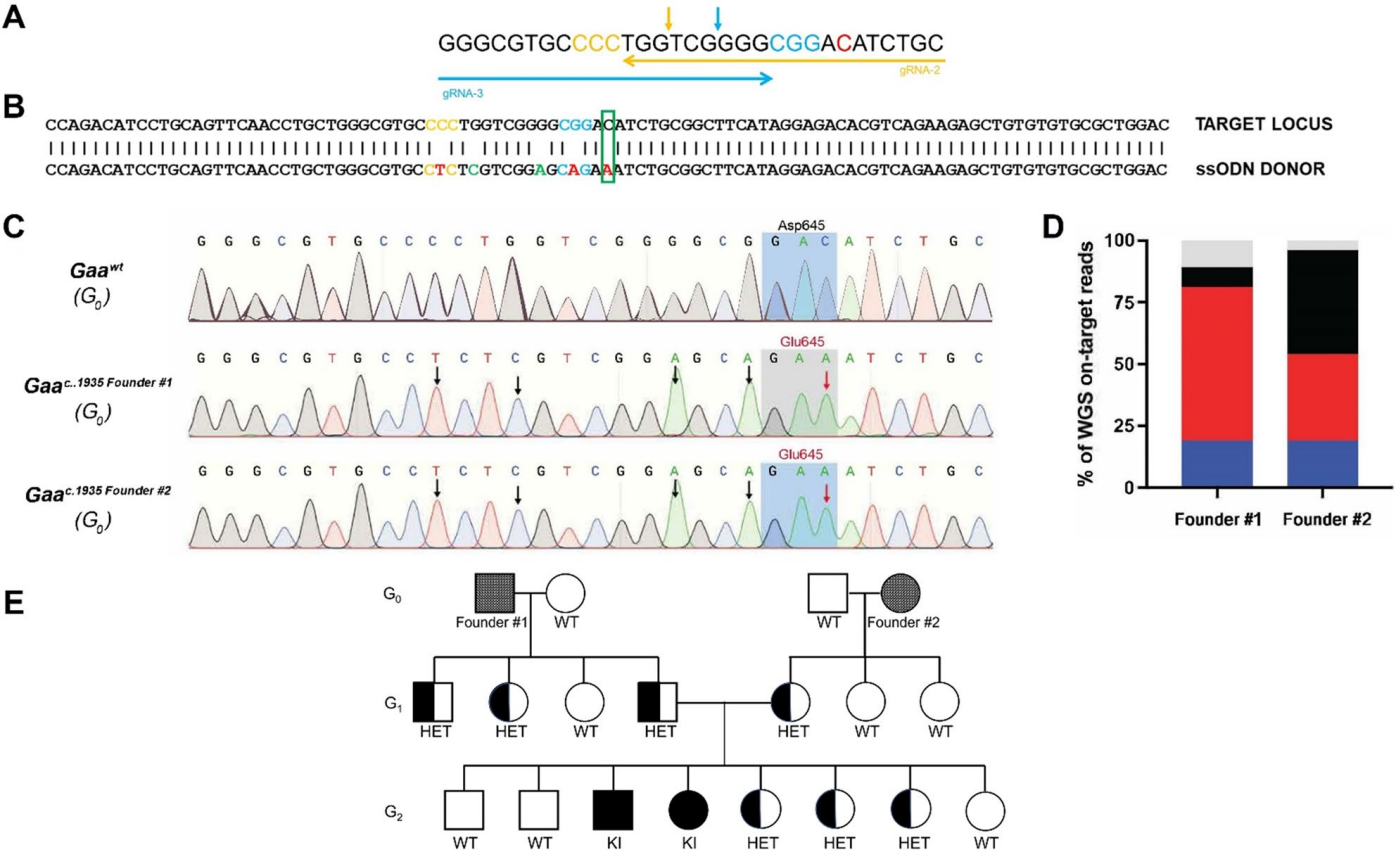
Generation of a *Gaa*^*em1935C*>*A*^ transgenic mouse line. (**A**) Dual overlapping guide RNA approach targeting the *Gaa*^*c.1935*^ target locus. Arrowhead direction indicates whether guide RNA is sense (right) or antisense (left). PAM sequences (NGG) are highlighted in color corresponding to the guide RNA arrows. The *Gaa*^*c.1935*^ locus for target adenine to cytosine nucelotide transversion is highlighted in red. Expected Cas9 nuclease cut sites are shown as vertical arrows in color corresponding to the guide RNA arrows. (**B**) Sequence of the target locus for integration (top) aligned with the ssODN (bottom) to introduce the *Gaa*^*c1935C*>*A*^ mutation (green box). PAM motifs are indicated in gold (gRNA-2) or blue (gRNA-3). Installed synonymous variants at PAM sites (*Gaa*^*c.1920C*>*T*^*, Gaa*^*c.1932G*>*A*^) and the desired KI mutation are highlighted in red. Installed gRNA seed region variants (*Gaa*^*c.1923G*>*C*^*, Gaa*^*c.1929G*>*A*^) are highlighted in green. (**C**) Sequencing chromatograms of control (*Gaa*^*wt*^), founder #1 (*Gaa*^*c.1935 Founder #1*^), and founder #2 (*Gaa*^*c.1935 Founder #2*^). Black arrows indicate synonymous variant edits at PAM sites (*Gaa*^*c.1920*^, *Gaa*^*c.1932*^) or gRNA seed regions (*Gaa*^*c.1923*^*, Gaa*^*c.1929*^). Red arrows indicate the desired KI mutation (*Gaa*^*c.1935C*>*A*^). Gray shaded region indicates amino acids at position 645 for each mouse. (**D**) WGS analysis (> 50 × read depth) of the *Gaa*^*c.1935*^ locus in G_0_ founder #1 and G_0_ founder #2. WGS analysis demonstrates highly efficient on-target genome-editing in these founder mice. Data are presented as stacked bar graphs indicating the percentage of WGS reads for each event category. Gray: nonspecific *Gaa* mutations; black: no *Gaa* mutation; red: intended *Gaa*^*c.1935C*>*A*^ mutation and associated synonymous variants; and blue: *Gaa* insertion/deletions. (E) Pedigree diagram of mating scheme to segregate the intended *Gaa*^*em1935C*>*A*^ KI allele from mosaic CRISPR-generated founder mice for generation of homozygous *Gaa*^*em1935C*>*A*^ KI mice. Males are represented as squares and females are represented as circles.

Given our prior success in generating *Gaa*^*em1826dupA*^ KI cell and transgenic mouse lines using a bi-directional, dual overlapping gRNA strategy^15^, an additional gRNA-3 (5′-GGGCGTGCCCCTGGTCGGGG-3′) was introduced (Fig. 2A). Comparing gRNA-3 with gRNA-1 by in silico analysis*,* gRNA-3 had higher predicted on-target efficiency (0.5487 [gRNA-3] vs 0.3905 [gRNA-1]) by CRISPick^13^ as well as lower predicted off-targets by GT-Scan^14^. We then applied the dual overlapping gRNA strategy in vivo using *Gaa*^*c.1935*^ gRNA-2 and gRNA-3 with the ssODN (Fig. 2B) via pronuclear injection of C57BL/6NJ single-cell embryos by standard methods^16^. 566 oocytes were injected, 531 oocytes (94.8%) were implanted, and a total of 39 founder mice were generated. The dual overlapping gRNA method achieved a high percentage of on-target editing activity in genome-edited founder mice (89.7%; 35 out of the 39 mutant mice) showing significant Cas9 activity/insertion/deletion (indel) mutations within the target region in *Gaa*. Among the founders, 15 mutants (38.5%) exhibited on Sanger sequencing the desired c.1935C>A KI mutation (See Table 2) along with the silent PAM and seed region mutations (Fig. 2C). Of these 15, 13 had significant (> 40% estimated by Sanger sequencing) indel mutations in the *Gaa* target region. Founder #1 (> 50% for *Gaa*^*c.1935C*>*A*^) and founder #2 (> 25% for *Gaa*^*c.1935C*>*A*^) had the highest percentage of the c.1935C>A mutation and lowest percentage of indels. The two founder mice and *Gaa*^*wt*^ mice underwent whole genome sequencing (WGS) at > 50 × coverage and on-target locus alignment analysis to better quantitate the extent of genomic mosaicism. For on-target analysis, *Gaa*^*c.1935*^ target loci from aligned FASTQ reads were designated to four categories: *Gaa*^*c.1935*^ KI mutation; indel mutation; no mutation; and nonspecific mutation. WGS analysis demonstrated highly efficient integration of the desired *Gaa*^*c.1935C*>*A*^ KI mutation with indel and nonspecific mutations comprising a minority of genomic editing events in *Gaa*^*c.1935C*>*A*^ founder mice (Fig. 2D). For off-target analysis of the WGS data, first we examined the seven genomic regions (Supplementary Table 1) predicted by GT-Scan as potential off-target sites of gRNA-2 and gRNA-3, and the only result was the intended *Gaa*^*c.1935C*>*A*^ mutation. No single nucleotide variations (SNVs) were detected within 500 bp of these sites. Next, we examined the founders’ WGS data for any de novo (compared to WT WGS) C>A transversions with a de novo N > A mutation 3 or 6 bases upstream, an N>C mutation 12 bases upstream, or an N>T mutation 15 bases upstream of the C>A suggesting ectopic SpCas9/HDR activity. No ectopic HDR signatures were identified in the genomes of either founder #1 or founder #2.

**Table 2:** Dual overlapping gRNA strategy and outcomes of *Gaa*^*em1935C*>*A*^ mouse generation.

Dual gRNA strategy components	Concentration	Founder mice with any *Gaa* mutation (% Positive)	Founder mice with *Gaa*^*em1935C*>*A*^ mutation (% Positive)
*Gaa*^*c.1935*^ crRNAs (gRNA-2, gRNA-3)	3 µM RNP complex	89.7% (35 of 39 founders)	38.5% (15 of 39 founders)
tracrRNA			
3xNLS SpCas9			
Donor ssODN	10 ng/µL		

Dual overlapping gRNA components and concentrations used in pronuclear microinjection of C57BL/6NJ fertilized zygotes are outlined. Each crRNA was hybridized with tracrRNA at a 1:1 ratio to form gRNA duplexes. Equimolar amounts of gRNAs were then combined with 3xNLS SpCas9 at a 1:1 ratio to form the RNP complex. Numbers of founder mice positive for any *Gaa* mutation and founder mice with the *Gaa*^*c.1935*^ KI mutation are reported as percentages.

Founder #1 and founder #2 were mated with WT animals, and their offspring G_1_ HETs (male from founder #1 and female from founder #2) were further crossed to obtain the first homozygous c.1935C>A KI mice in the G_2_ generation (Fig. 2E). Subsequently, mice harboring the c.1935C>A *Gaa* variant were backcrossed 10 generations to the C57BL/6NJ background before KI mice were characterized. As the generation of our KI mice involved CRISPR endonuclease-mediated mutation introduction, we followed the International Committee on Standardized Genetic Nomenclature for Mice^17^ and named the KI transgenic mice as *Gaa*^*em1935C*>*A*^.

### *Gaa*^*em1935C*>*A*^ KI mice have severe GAA enzymatic deficiency and glycogen storage in cardiac, skeletal muscle, and brain tissue

**Figure 3:**
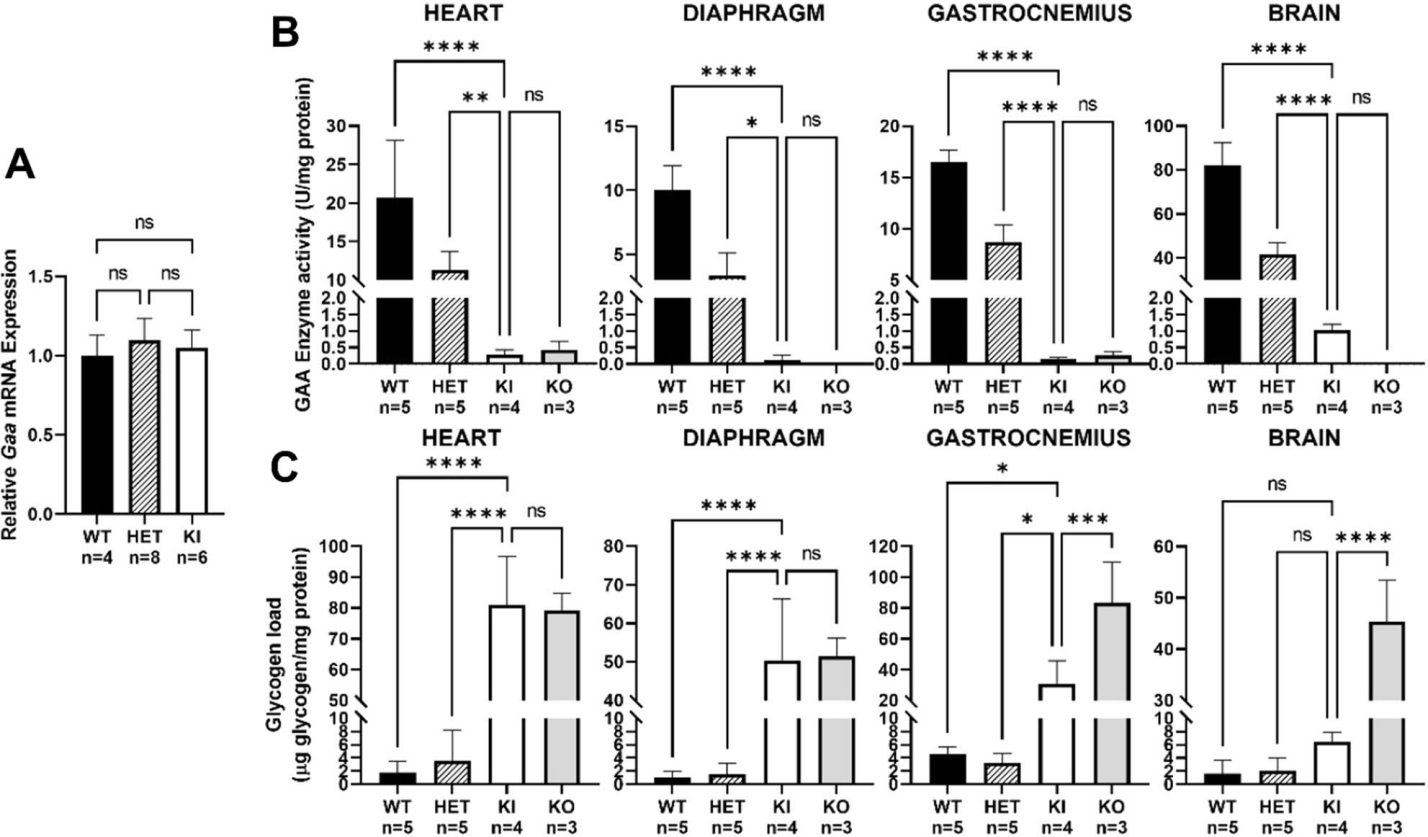
Molecular and biochemical characterization of *Gaa*^*em1935C*>*A*^ KI mice. (**A**) *Gaa* mRNA expression in tail or liver biopsy samples from 3-month-old WT (n = 4; black bar), HET (n = 8; striped bar), and KI (*Gaa*^*em1935C*>*A*^; n = 6; white bar) mice *Gaa* expression levels were measured by TaqMan probe-based quantitative real-time PCR using the ΔC_t_ method for comparison of the target gene (*Gaa*) to the reference gene (*Gapdh*). The average C_t_ value from WT samples were further utilized to normalize with other groups. No significant difference in *Gaa* mRNA transcript expression was detected among WT, HET, and KI samples. (**B**) GAA enzyme activity in heart, diaphragm, and gastrocnemius muscle tissues and brain homogenate from WT (n = 5; black bars), HET (n = 5; striped bars), KI (*Gaa*^*em1935C*>*A*^; n = 4; white bars), and KO (*Gaa*^*tm1Rabn*^; n = 3; grey bars) mice was measured using a fluorometric 4-MU α-d-glucopyranoside assay and normalized to the amount of sample protein. (**C**) Glycogen level was measured in the same tissues used for analysis in (**B**) using a colorimetric assay. KO mice displayed significantly elevated glycogen levels relative to WT and HET mice in all tissues assayed. However, KI mice showed a significant elevation of glycogen levels in muscle tissues, but no significant elevation in brain. The amount of glycogen was normalized to the amount of sample protein. Data were generated from at least three independent experiments and shown as mean ± SD. All comparisons were analyzed using one-way ANOVA with the Tukey post-hoc test. *p < 0.05, **p < 0.01, ***p < 0.001, ****p < 0.0001. ns: not significant.

The missense *Gaa*^*c.1935C*>*A*^ mutation in exon 14 of the *Gaa* gene leads to an amino acid substitution; therefore, we did not expect any nonsense-mediated decay in *Gaa*^*c.1935C*>*A*^ mRNA transcripts. The comparative ΔC_t_ between mouse *Gaa* and housekeeping gene *Gapdh* acquired by RT-PCR among WT, HET, and KI groups are almost identical, indicating the *Gaa*^*c.1935C*>*A*^ mutation does not affect *Gaa* mRNA levels (Fig. 3A).

GAA enzymatic activity was measured with artificial fluorometric 4-MU substrate as described previously^15^. The results were consistent with the other findings from this study, showing that the HET group had close to 50% of the level of enzymatic activity observed in the WT group in each muscle tissue and brain tissue sample tested, indicating that the one WT allele produced functional enzyme, but not the c.1935C>A allele. For comparative purposes, we acquired *Gaa* homozygous knock-out (KO) (B6;129-*Gaa*^*tm1Rabn*^/J; exon 6 knock-out)^18^ mouse tissues from Jackson Laboratory (Bar Harbor, ME). Compared to tissue from WT or HET animals, tissue from KI (*Gaa*^*em1935C*>*A*^) and KO (*Gaa*^*tm1Rabn*^/J) animals had significantly decreased GAA enzymatic activity (about 1% of WT levels) (Fig. 3B).

Compared to the unaffected WT or HET groups, KI and KO mice had abnormally elevated lysosomal glycogen storage in heart, diaphragm, and gastrocnemius muscle tissue. Interestingly, increased glycogen storage in whole-brain homogenates was observed in KO mice, but not in KI mice, which had a slight, but not statistically significant, increase in glycogen load (Fig. 3C).

### *Gaa*^*em1935C*>*A*^ KI mice show increased muscle glycogen content and elevated LAMP1 marker in brain regions

**Figure 4:**
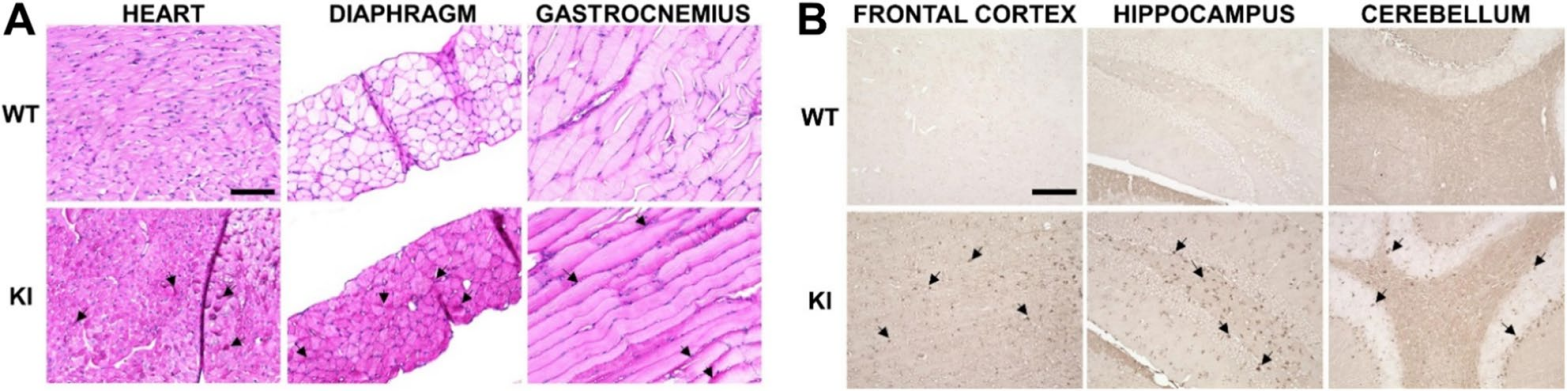
Tissue pathology in *Gaa*^*em1935C*>*A*^ KI mice showing glycogen storage in muscles and lysosomal abnormality in brains. (**A**) Representative bright-field images of heart, diaphragm, and gastrocnemius sections from 3-month-old WT and KI mice, stained with hematoxylin/PAS. Areas of abnormal glycogen accumulation (arrowheads) in cardiac and skeletal muscle tissues were observed in KI mice compared to WT mice (top). (**B**) Immunohistostaining with mouse anti-LAMP1 antibody showing increased cell body staining (arrowheads) in frontal and hippocampal neurons and Purkinje cells of KI mice from all three representative brain areas (frontal cortex, hippocampus and cerebellum). Scale bar represents 100 µm.

PAS staining is routinely used to demonstrate abnormal carbohydrate accumulation in muscle tissue^19^. PAS staining was performed in different muscle tissues (heart, diaphragm, and gastrocnemius) from 3-month-old KI mice. Scattered red to magenta PAS staining particles representing the accumulation of glycogen were observed in all three muscle tissue types in the KI mice, but not in WT animals (Fig. 4A). PAS staining with diastase (PAS-D), an enzyme that digests only glycogen, was also applied to consecutive slides to confirm that the particles consisted of glycogen. A decrease in red/magenta signal confirms that excessive accumulation products in tissues comprised only glycogen (Supplementary Fig. 1).

The lysosomal associated membrane protein-1 (LAMP1) is commonly used as a biomarker for lysosomal storage. LAMP1 staining in the brain sections from 3-month-old WT and KI mice were examined in three representative areas of the brain (frontal cortex, hippocampus, and cerebellum), demonstrating markedly increased LAMP1 immunoreactivity in KI neuronal cell bodies compared to WT controls (Fig. 4B).

In summary, histopathology showed that the KI mice display early pathological glycogen accumulation in muscle tissues, which is analogous to muscle pathology in IOPD patients. In addition, the KI mice display a more pronounced lysosomal burden in the brain areas as early as 3-months of age compared to WT animals.

### *Gaa*^*em1935C*>*A*^ KI mice have impaired skeletal muscle autophagy

Excessive autophagic buildup is well-documented in PD patients and in PD mice^20,21^ and may be a potential mechanism of PD pathogenesis. Microtubule-associated protein light chain 3 (LC3B) is a protein component of autophagosomes, which are quickly degraded under normal physiological conditions and are hardly detectable. Cleavage of LC3B at the carboxy terminus immediately following synthesis yields the cytosolic, non-autophagosome bound LC3B-I form. LC3B-I is converted to autophagosome-bound LC3B-II via conjugation to phosphatidylethanolamine when autophagic processes are activated. Following autophagosome-lysosome fusion, LC3B-II is then hydrolyzed back to LC3B-I via ATG5^22^.

**Figure 5:**
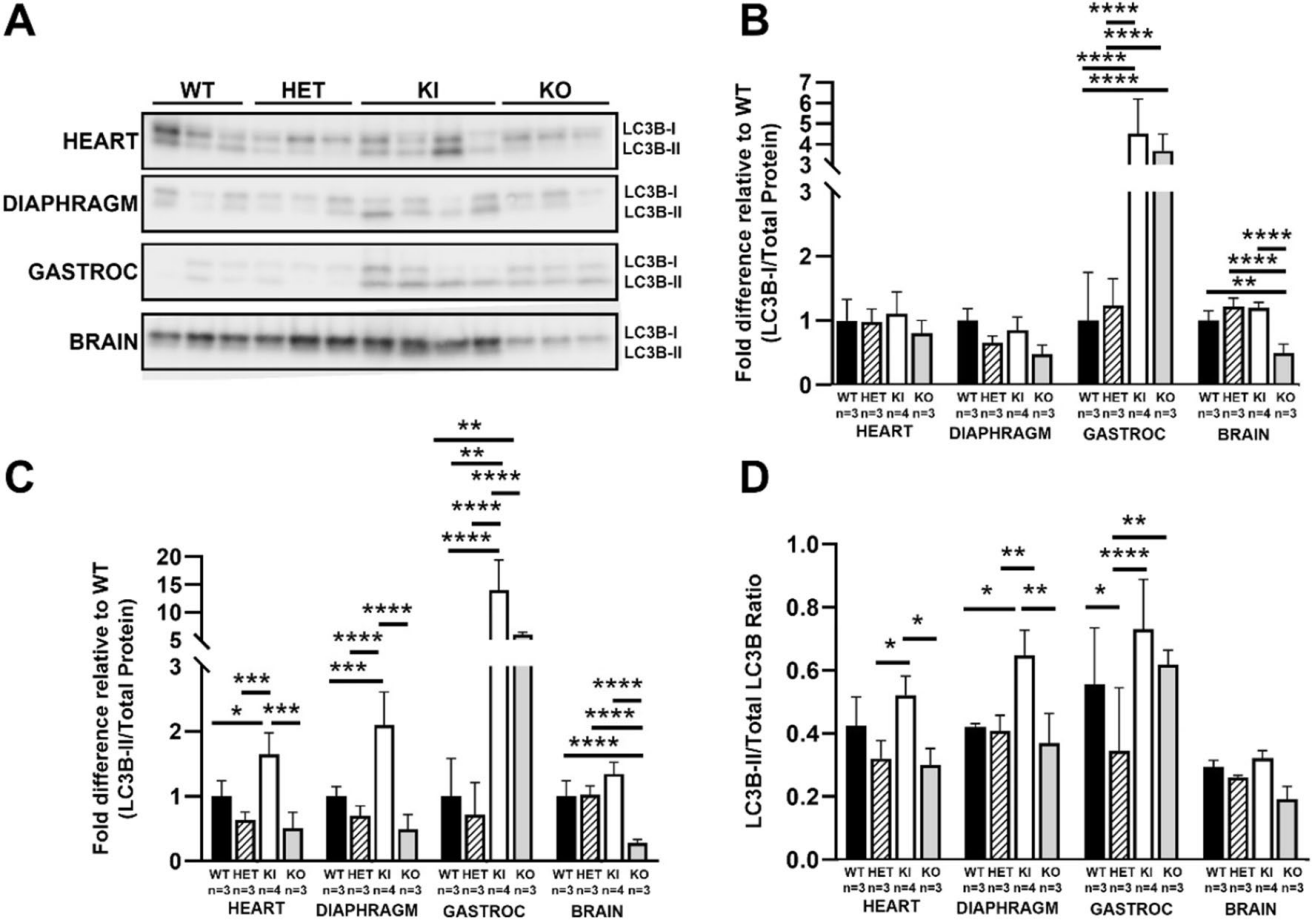
Autophagy impairment in the *Gaa*^*c.1935C*>*A*^ KI mouse model. (**A**) Representative western blot images of autophagy-associated proteins (LC3B-I and LC3B-II) from tissue homogenate from heart, diaphragm, gastrocnemius, and brain of WT (n = 3; black bars), HET (n = 3; striped bars), KI (n = 4; white bars), and *Gaa*^*tm1Rabn*^ (KO, n = 3; grey bars) mice. Prominent LC3B-II bands can be seen in KI and KO tissues. (**B**) LC3B-I and (**C**) LC3B-II protein levels normalized to the amount of total protein. Cytosolic LC3B-I is markedly elevated in KI and KO gastrocnemius muscle; autophagosomal LC3B-II is markedly elevated in KI and KO gastrocnemius, and moderately elevated in KI heart and diaphragm. (**D**) LC3B-II/LC3B-I ratio normalized to WT. Impaired autolysosomal formation (increased LC3B-II/LC3B-I ratio) is observed in KI skeletal muscle tissues. The ratio of LC3B-II and LC3B-I protein intensity was quantified by densitometric analysis of the western blots, and the ratio was further normalized with WT in each tissue assayed. Data were generated from at least three independent western blots and values are shown as mean ± SD. All comparisons were analyzed using one-way ANOVA with the Tukey post-hoc test. *p < 0.05, **p < 0.01, ***p < 0.001, ****p < 0.0001.

To examine autophagic status of the *Gaa*^*em1935C*>*A*^ KI mice, western blotting for LC3B was performed using tissue homogenate (Fig. 5A and Supplementary Fig. 2). Both KI and KO models demonstrate elevated synthesis of LC3B-I in gastrocnemius, evidence of upregulated autophagy (Fig. 5B); further, autophagosomal LC3B-II is increased in KI heart, diaphragm, and gastrocnemius but not in brain (Fig. 5C). The ratio of LC3B-II:LC3B-I is increased (Fig. 5D), demonstrating impaired autophagosome-lysosome fusion, in skeletal muscles (diaphragm and gastrocnemius) but not cardiac muscle of the KI model. This is an observation similar to what has been observed in both *Gaa*^*em1826dupA*^ KI and KO mouse models^15,21^.

### *Gaa*^*em1935C*>*A*^ KI mice display left ventricular cardiac hypertrophy at 3 months of age

**Figure 6:**
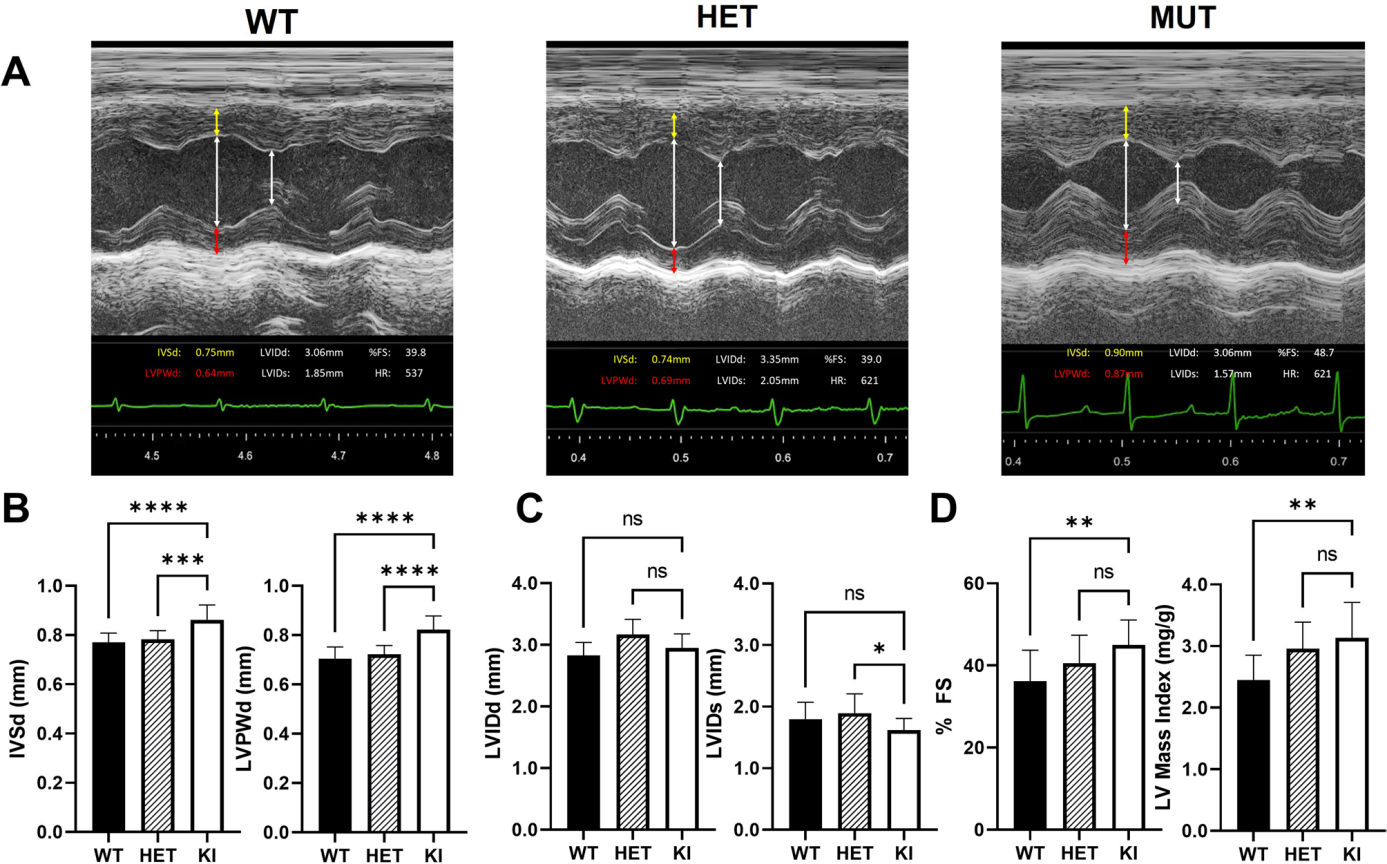
Three-month-old *Gaa*^*em1935C*>*A*^ KI mice display anatomical features of left ventricular cardiac hypertrophy. (**A**) Representative M-mode echocardiographic images showing cardiac dimensions: IVSd: yellow arrows; LVPWd: red arrows, and LVIDd / LVIDs: white arrows. (**B**) KI IVSd and LVPWd are significantly increased versus WT and HET animals, indicative of concentric hypertrophic cardiomyopathy. (**C**) LVIDd and LVIDs do not significantly differ between WT, HET, and KI animals. (**D**) KI fractional shortening is significantly increased versus WT indicative of hyperdynamic contractility; KI LVMI is significantly increased versus WT indicative again of hypertrophic cardiomyopathy. All measurements from WT (n = 12; black bars), HET (n = 10; striped bars), and KI (*Gaa*^*.em1935C*>*A*^; n = 10; white bars) mice were obtained from 3-month-old mice. Data are shown as mean ± SD. Heart rate (HR) was maintained greater than 500 bpm throughout measurements. All comparisons were analyzed using one-way ANOVA with the Tukey post-hoc test. *p < 0.05, **p < 0.01, ***p < 0.001, ****p < 0.0001. ns: not significant.

Neonatal-onset hypertrophic cardiomyopathy is a common clinical presentation in patients with IOPD. To explore the anatomical features and physiological function of hearts in the KI mice, echocardiography was performed on 3-month-old mice. M-mode images obtained by echocardiography were used to measure multiple parameters including wall thickness, internal diameter, and heart rate. Many additional functional parameters can be derived from these measurements to determine temporal left ventricular (LV) wall motion as an index for LV contractile patterns and chamber size (Fig. 6A).

Increases in interventricular septal diameter (IVSd), LV posterior wall diameter (LVPWd), and LV mass index (LVMI) were observed in KI mice, compared to WT and/or HET mice (Fig. 6B), indicating pronounced hypertrophic cardiomyopathy. Measurements of myocardial contraction showed a slight decrease in LV systolic internal diameter (LVIDs) in the KI mouse, but no significant difference in LV diastolic internal diameter (LVIDd) was observed among WT, HET, and KI mice (Fig. 6C). Increased fractional shortening indicative of cardiac contractile dysfunction was observed in KI mice (Fig. 6D). Echocardiographic data therefore indicates early hypertrophic cardiomyopathy phenotypes in 3-month-old *Gaa*^*em1935C*>*A*^ KI mice. The data presented in Fig. 5 show no gender differences in these parameters (Supplementary Fig. 3).

### Reduced forelimb grip strength in *Gaa*^*em1935C*>*A*^ KI mice

The forelimb grip strength test is commonly used to evaluate neuromuscular dysfunction in mice by measuring the deterioration of skeletal muscle. Peak tension force was recorded as the mice lost their grip on the force transducer bar and normalized to bodyweight for analysis by gender group.

**Figure 7:**
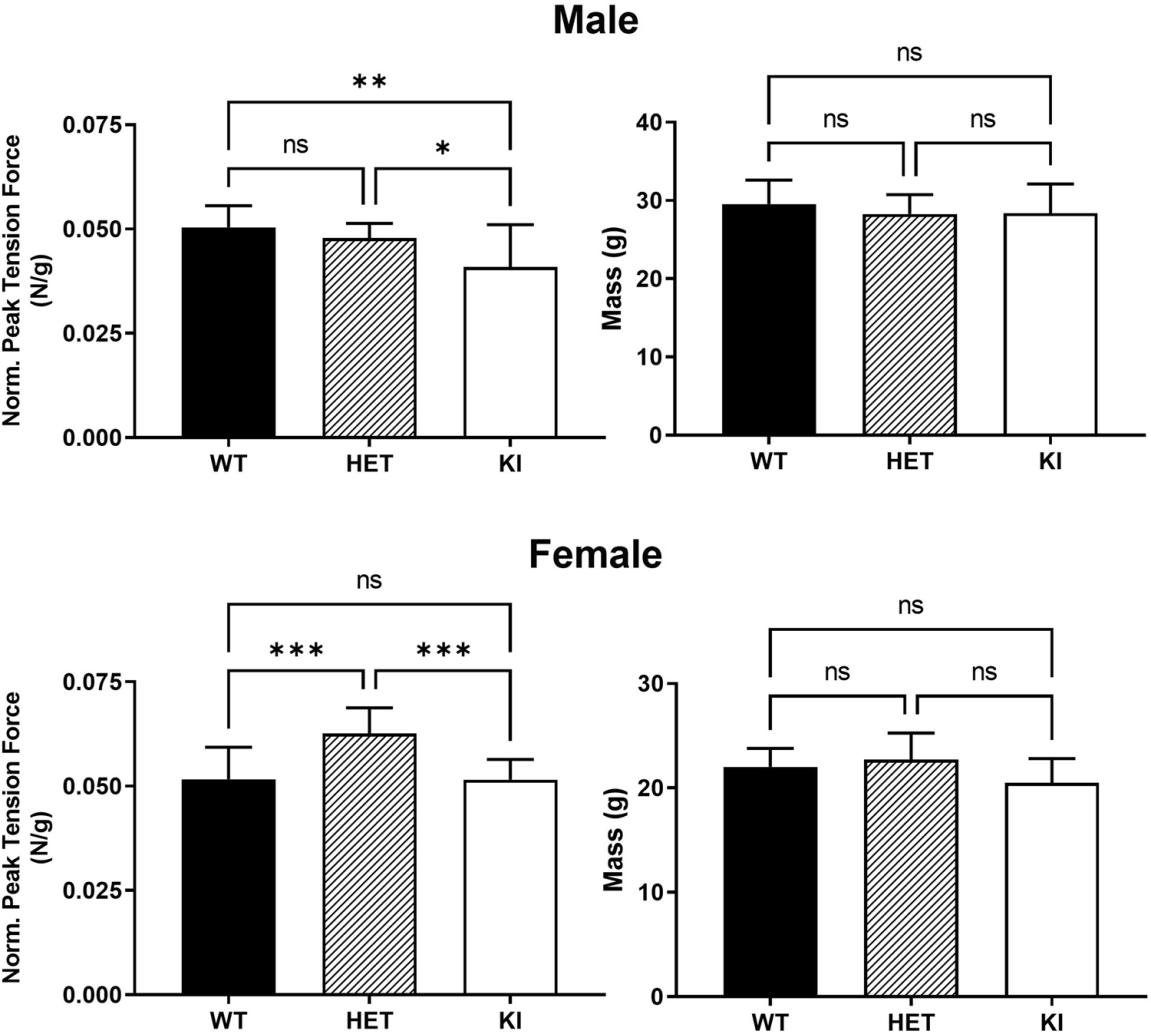
Reduced forelimb grip strength in male *Gaa*^*em1935C*>*A*^ transgenic mice. Forelimb peak tension force and body mass measurements in 3-month-old male WT (n = 12; black bars), HET (n = 12; striped bars), and KI (n = 14; white bars) mice (top panel) and female WT (n = 12; black bars), HET (n = 12; striped bars), and KI (n = 11; white bars) mice (bottom panel). No significant difference in body weight in each gender cohort among WT, HET and ki groups. Male KI mice demonstrate decreased normalized peak tension force consistent with skeletal muscle weakness. Forelimb peak tension force was measured using a grip strength meter and taken as the average of 9 trials over 3 days. Data are shown as mean ± SD. All comparisons were analyzed using one-way ANOVA with the Tukey post-hoc test. *p < 0.05, **p < 0.01, ***p < 0.001. ns: not significant.

First, mouse body weight is known to differ between genders at 3 months of age^23^. The mean ± SD body weights of male and female mice in our study cohort were 28.73 ± 3.13 g and 21.76 ± 2.36 g, respectively. In each gender cohort, there was no significant difference in body weight across WT, HET, and KI mice (Fig. 7). In addition, at 3 months of age, the male *Gaa*^*em1935C*>*A*^ KI mouse showed a significant reduction (~ 19%) in normalized peak tension force compared to WT mice, indicating decreased forelimb muscle strength in KI mice (Fig. 7). This reduction was observed only in male KI mice, but not in female KI mice.

## Discussion

In populations of Southern Han ancestry, the *GAA* c.1935C>A (p.Asp645Glu) mutation represents 36%-80% of mutations^11,12,24^ in IOPD patients. We have successfully applied CRISPR/Cas9 genome editing to install the *Gaa* c.1935C>A mutation in a mouse myoblast C2C12 cell line and create a novel *Gaa*^*em1935C*>*A*^ KI mouse model; each of which represents a valuable resource for studying IOPD. The KI C2C12 line demonstrates severe GAA enzyme deficiency and glycogen accumulation; the KI mouse model successfully recapitulates molecular, biochemical, histologic, and phenotypic aspects of human IOPD.

While no phenotypic differences were noted between *GAA* c.1935C>A HET and WT mice aside from the expected 50% reduction in HET GAA enzymatic activity, the homozygous KI mice demonstrated a significant, PD-like phenotype. KI mice had normal *Gaa* mRNA levels with significantly reduced level of GAA hydrolysis activity (about 1% of WT) in heart and skeletal muscle, as well as brain tissue. This aligns with observed levels of low GAA enzyme activity (0.08–0.82% of normal range for control) previously measured in homozygous *GAA* c.1935C>A patient fibroblasts^25^. Significant increases in glycogen storage were observed in KI mouse muscle tissues, consistent with the human *GAA* c.1935C>A IOPD phenotype. In addition, increased lysosomal burden, as indicated by LAMP1 immunostaining, was demonstrated in brain tissue from *Gaa*^*em1935C*>*A*^ KI mice. Autophagic impairment was noted in skeletal muscle tissues, consistent with what is observed in human PD and other murine PD models^18^. *Gaa*^*em1935C*>*A*^ mice developed hypertrophic cardiomyopathy at approximately two months of age, which becomes quite marked at three months of age. This muscle weakness phenotype may be due to a combination of sequelae from cardiomyopathy, impairment of lysosomal-autophagosomal fusion into autolysosomes, and catabolism of myofibril contractile proteins^22^. Studies are ongoing to assess the life span, natural history, and phenotypic progression of the model.

A significant divergence of the model from human *GAA* c.1935C>A IOPD is the lack of infantile mortality in KI mice. This KI mouse, along with the *Gaa*^*em1826dupA*^ KI mouse strain previously generated in our laboratory^15^ and other previously published *Gaa* KO models^18,26,27^, all demonstrate null or nearly-zero GAA enzyme activity. Nevertheless, no neonatal mortality has been observed in any model, while neonatal death is the inevitable clinical outcome in untreated IOPD patients^28,29^. Only one *Gaa* KO model on a DBA/2J background (homozygous *Ltbp4*^*Δ36*^ alleles) is reported to have a shorter lifespan (but still not neonatal lethality) in male mice, compared to male *Gaa* KO mice on the C57BL/6;129 background^27^. The DBA/2J genetic background may exacerbate the severity of respiratory muscle weakness caused by *Gaa* KO deletion, leading to earlier death than is observed in other KO models^27^.

Genome editing represents a new approach to the treatment of PD, compared to traditional treatments like ERT or gene therapy. A mouse that both recapitulates clinical features of human disease and harbors orthologous pathologic gene variants serves as a valuable system for the development of innovative therapies and, most importantly, studies enabling eventual clinical trials in humans. As this model undergoes in-depth validation and studies of its clinical and immune response to standard intravenous rhGAA enzyme infusions, subsequent avenues for exploration include variant rhGAA enzyme infusions, gene therapy, and CRISPR-based genomic editing. The latter approach can be performed using CRISPR “prime editing”, which is capable of targeting more than 90% of known pathogenic mutations, including the c.1935C>A transversion^30^. In addition, multiple tissues can be obtained or derived from our *Gaa*^*em1935C*>*A*^ KI mouse to investigate the potential tissue-specific efficacy of genome correction-based therapeutics in vitro, before in vivo studies are attempted. With these advances, and high sequence conservation surrounding the mutation, the *Gaa*^*em1935C*>*A*^ KI mouse represents an ideal candidate for the development of personalized therapeutics like prime editing that correct pathogenic variants, restore GAA enzyme activity and further improve functional phenotypes before translational application in the clinic.

## Materials and methods

### ***Gaa***^***c.1935***^ guide RNA SpCas9 expression vector cloning

All oligonucleotides applied in this project were manufactured by Integrated DNA Technologies (Coralville, IA). Guide RNA (gRNA) oligonucleotides with *Bbs*I (New England Biolabs) restriction enzyme overhangs were designed with forward oligo (5′-CACCG(gRNA)-3′) and reverse oligo (5′-AAAC(reverse complement gRNA)C-3′). Complementary gRNA oligonucleotides were cloned into pSpCas9(BB)-2A-Puro plasmid (pX459; Addgene plasmid ID# 48139) using the *Bbs*I site. Positive pX459-gRNA clones were confirmed by Sanger sequencing and further expanded using the PureLink HiPure Plasmid Midiprep Kit (Invitrogen). All donor ssODNs were designed with 50-bp homology arms flanking the target locus and synonymous mutations in the PAM and seed region (5 nt upstream of PAM) to prevent further Cas9 activity after successful HDR.

### In vitro testing of *Gaa*^*c.1935*^ guide RNAs

pX459-*Gaa*^*c.1935*^ gRNA expression vectors and donor ssODNs were transfected into murine C2C12 myoblast cells (ATCC CRL-1772) using the Neon™ Transfection System (ThermoFisher Scientific) as previously described^15^. In short, 3 × 10^5^ cells were mixed with 4.5 μg gRNA expression vector(s) and 450 nM ssODN (Table 1), then and electroporated using the following parameters: pulse voltage, 1650 V; pulse width: 10 ms; pulse number: 3. Forty-eight hours post-transfection, cellular genomic DNA was obtained for Sanger sequencing around the *Gaa*^*c.1935*^ target locus. SpCas9 nuclease activity and HDR KI efficiency were determined by Tracking of Indels by Decomposition (TIDE)^31^ or Tracking of Insertion, Deletions, and Recombination events (TIDER)^32^ analysis of DNA sequence electropherogram files.

### Generation of a *Gaa*^*c.1935C*>*A*^ KI C2C12 cell line

Similar parameters to those described above were applied to transfect 4.5 μg of gRNA-1, gRNA-2 under the U6 promoter in the pX459 expression vector and 450 nM ssODN into 3 × 10^5^ C2C12 myoblast cells. pCMV6-AC-GFP (OriGene) was used as a marker for transfection; electroporated cells were selected for successful pX459 transfection by adding 2.5 μg/mL puromycin dihydrochloride (Sigma-Aldrich) to the culture medium beginning 24 h after electroporation. Puromycin was supplied every 48 h until all pCMV6-AC-GFP-transfected cells were no longer viable. After puromycin-resistance screening, single cell clones were selected by standard serial dilution methods in 96-well plates in the presence of 2.5 μg/mL puromycin dihydrochloride. Sanger sequencing was used to confirm the genotype of each single cell clone.

### Generation of *Gaa*^*em1935C*>*A*^ KI mice

The generation of *Gaa*^*em1935C*>*A*^ KI mice was performed at the University of California-Irvine Transgenic Mouse Core, and all study procedures were reviewed and approved under IACUC protocol #AUP16–63. Standard methods were applied to produce pronuclear stage C57BL/6NJ embryos^16^. In brief, 3 μM crRNA/tracrRNA/3xNLS-Cas9 protein and 10 ng/μL ssODN were injected into pronuclear stage C57BL/6NJ embryos (Table 2). Surviving embryos were implanted into oviducts of 0.5dpc ICR pseudo-pregnant females.

### Whole-genome sequencing and analysis

Whole genome sequencing (WGS) and analyses were performed on tail samples from G_0_ wild type (*Gaa*^*wt*^), G_0_ founder #1 (*Gaa*^*c.1935Founder#1*^), and G_0_ founder #2 (*Gaa*^*c.1935Founder#2*^) mice. In brief, WGS was performed and analyzed on an Illumina HiSeq X Ten Sequencer at 40–50× read depth (Fulgent Genetics) using TrueSeq DNA libraries created from 1 μg fragmented genomic DNA. WGS on-target and off-target analyses were performed on the OnRamp BioInformatics platform. Data were aligned to the Mouse genome (mm10) using BWA^33^. PCR artifacts were identified with the memtest utility from Sentieon^34^, and filtered out using samtools^35^. Alignments were de-duplicated and realigned around insertions and deletions using LocusCollector, Dedup, and Realigner from Sentieon. SNV calling was performed with GVCFtyper from Sentieon, using the mouse dbSNP 142 data (http://hgdownload.cse.ucsc.edu/goldenpath/mm10/database/snp142.txt.gz) as the known SNPs. Known SNPs and variants falling in un-located chromosomes were removed from analysis.

For off-target analysis, we used SNVs that had a C>A transversion and any one of the four following criteria indicating an ectopic HDR event: a de novo N → A mutation 3 bases upstream, N→A mutation 6 bases upstream, N→C mutation 12 bases upstream, or N → T mutation 15 bases upstream. This search step was repeated for the reverse complement sequences. The fully processed BAM files (after Realigner) were used as input to the Manta structural variant caller^36^. For each of the non-wild-type (WT) samples, Manta somatic caller was applied with the C57BL6-WT sample as “normal” and the sample of interest as “tumor,” thereby subtracting the background structural variants in C57BL6-WT compared to mm10. Vcf (https://vcftools.github.io) was used to annotate the output VCF files from Manta.

### Experimental animals

The G_2_ mice were backcrossed 10 generations onto a C57BL/6NJ background before any characterization was performed. Mice received ad libitum Teklad Global 16% Protein Rodent Diet (Envigo, Indianapolis, IN) and water in temperature-controlled environment. Animal were housed in groups of 4 mice/cage, separated by gender except for mating trios, and provided with 14-h light and 10-h dark cycle. The use and care of animals in this study adhered to the guidelines of the NIH Guide for the Care and Use of Laboratory Animals, as utilized by the CHOC Children’s Institutional Animal Care and Use Committee under CHOC IACUC protocol #160,902. In addition, all experiments in this study were carried out in compliance with ARRIVE guidelines (https://arriveguidelines.org), and all methods were performed in accordance with relevant guidelines and regulations.

Genotyping was performed by Sanger sequencing to confirm the *Gaa*^*c1935*^ target locus with the following primers: *GAA*_c1935(F), 5′- CAGGCGTTAGGACAAATGGA-3′; *GAA*_c1935(R), 5′- TTCCAGCAGGTATGGGATTAAC-3′. Heterozygous (*Gaa*^*wt/em1935C*>*A*^) males and females were crossed to obtain homozygous KI, heterozygous (HET), and WT mice for this study. Experiments were performed on age-matched mice of either gender (usually littermates). Homozygous knock-out (KO) (B6;129-*Gaa*^*tm1Rabn*^/J)^18^ mouse tissues for comparative molecular and biochemical analyses were acquired from Jackson Laboratory (Bar Harbor, ME).

### Quantitative real-time PCR

Total RNA was extracted from tail tip or liver homogenate using a Direct-zol RNA miniprep kit (Zymo Research) and reverse-transcribed using an iScript™ cDNA Synthesis Kit (Bio-Rad). As per the manufacturer’s instructions, both oligo(dT) and random hexamer primers were used to synthesize cDNA. The resulting cDNA was diluted tenfold, and a 2-μl aliquot was used in a 12-μl PCR reaction with SsoAdvanced Universal Probes Supermix (Bio-Rad) and specific TaqMan primer/probe assays for *Gaa* (Taqman assay #Mm00484581_m1) and *Gapdh* (TaqMan assay #Mm99999915_g1). PCR reactions were run in triplicate and quantified with Bio-Rad CFX96 Touch Real-Time PCR Detection. *Gapdh* was used as an internal reference gene, and relative quantification of *Gaa* gene expression was calculated using the comparative ΔC_t_ method for the difference in C_t_ values of *Gaa* and *Gapdh* in the given sample. ΔC_t_ values were further normalized with the average of the C_t_ value of wildtype samples.

### Biochemical analyses

For the GAA activity assay, phosphate-buffered saline (PBS)-flushed mouse tissues or C2C12 myoblast cell pellets were homogenized in CelLytic M cell lysis reagent (MilliporeSigma). Acidic α-glucosidase enzyme activity was assessed as previously described with minor modifications^15,37^. In brief, 10 µL tissue homogenate was mixed with 10 µL of 6 mM 4-methylumbelliferyl-α-d-glucopyranoside substrate (MilliporeSigma) in McIlvaine citrate/phosphate buffer (pH 4.3) and quenched with 180 µL glycine carbonate buffer (pH 10.5) after 1-h incubation at 37 °C in a 96-well plate. GAA activity reactions were run in triplicate, and fluorescence measurements were obtained using an Infinite M Plex spectrofluorophotometer (Tecan) at excitation and emission wavelengths of 360 nm and 450 nm, respectively. One GAA enzymatic activity unit was defined as 1 nmol converted substrate per hour. Protein concentration was estimated using a Pierce BCA assay kit (ThermoFisher), using bovine serum albumin as a standard. Specific activity was calculated as units of GAA enzymatic activity per mg of protein.

Tissue glycogen levels were measured using a glycogen assay kit (Sigma-Aldrich) according to the manufacturer’s instructions. In brief, 10 µL tissue homogenate was incubated with hydrolysis enzyme reaction mixture in a final volume of 50 µL at room temperature for 30 min before adding 50 µL development enzyme reaction mixture for 30 min incubation at room temperature. Absorbance at 570 nm was measured using an Infinite M Plex spectrofluorophotometer (Tecan). A standard curve was generated using standard glycogen solution provided in the assay kit. Glycogen quantification assays were performed in duplicate, and an extra reaction without hydrolytic enzyme treatment was used for background correction of endogenous glucose levels in each sample. Tissue glycogen level is expressed as µg of glycogen per mg of protein.

### Tissue harvesting, processing, and histological staining

Three-month-old mice were euthanized using CO_2_ asphyxiation and transcardially perfused with PBS. Brains were dissected sagittally along the midline; left hemispheres were rapidly frozen and stored at − 80 °C for biochemical analysis, and right hemispheres were post-fixed at 4 °C in zinc formalin. Heart, diaphragm, and gastrocnemius muscle were also harvested. Half of the tissue samples for biochemical studies were rapidly frozen and the other half of tissues were post-fixed at 4 °C in zinc formalin.

Samples for histological staining were processed and embedded in paraffin blocks for sectioning at 4-μm thickness, and periodic acid-Schiff (PAS) staining (Sigma-Aldrich) was performed according to the manufacturer's instructions. EVOS M5000 imaging system (Invitrogen) was used to capture representative images at 20× objective magnification on RGB-mode illumination.

LAMP1 immunohistochemistry staining in paraffin-embedded brain sections from study animals were performed with anti-LAMP1 polyclonal antibody (Cat#24170, Abcam, Waltham, MA) using an ImmPACT DAB Substrate Kit with Peroxidase (Vector Laboratories, Burlingame, CA) following manufacturer’s instructions. Paraffin sections were deparaffinized and endogenous peroxidase activity was quenched by immersion in 1.5% hydrogen peroxide followed by heat-induced epitope retrieval in sodium citrate buffer. The sections were subsequently incubated overnight at 4 °C with mouse anti-LAMP1 antibody (1:50) following secondary antibody amplification before visualizing with diaminobenzidine (DAB) as chromogen. Represented images were captured by Keyence BZ-X800 microscopes (Keyence American, Itasca, IL) at 20× objective magnification with the same parameters of exposure.

### LC3B western blot analysis

Frozen mouse tissues were homogenized in CelLytic M cell lysis reagent (MilliporeSigma) and cOmplete protease inhibitors (Roche) was added to prevent protein degradation. Total protein concentration of the supernatants from centrifuged tissue lysates was determined by BCA protein assay (Pierce). Eight micrograms of total protein lysate were resolved on 4–15% Mini-PROTEAN TGX Stain-free gels (Bio-Rad) and transferred onto Immuno-Blot PVDF membranes (Bio-Rad). Membrane blots were blocked with EveryBlot blocking buffer (Bio-Rad) and probed with an anti-LC3B primary antibody (cat# L7543, Sigma) followed by an HRP-conjugated secondary antibody before applying ECL HRP substrate (Bio-Rad) for chemiluminescence. Stain-free gels and blots were imaged using the stain-free and chemiluminescence settings on the ChemiDoc™ MP imaging system (Bio-Rad). LC3B-I and LC3B-II protein levels were measured by densitometric analysis of western blots using Fuji software (ImageJ version 2.0)^38^. Signals were normalized to the amount of total protein as determined by densitometric analysis of stain-free gels. LC3B-II/LC3B-I ratio was normalized to WT for each organ.

### Murine echocardiography

Transthoracic echocardiography (M-mode and 2-dimensional echocardiography) was performed using a Vevo 2100 high-resolution ultrasound system, with a linear transducer of 32–55 MHz (VisualSonics Inc.). Chest fur was removed by using depilatory cream one day prior to the procedure. Mice were kept warm on a heated platform (37 °C) and anesthetized with 5% isoflurane delivered via nose cone for 15 s, then maintained at 0.5% throughout the echocardiography examination. Small needle electrodes for simultaneous electrocardiography were inserted into one upper and one lower limb. Measurements of chamber dimensions and wall thickness were performed while heartbeats of the mice were greater than 500 beats per minute (bpm). Percentage fractional shortening (%FS) was used as an indicator of left ventricular systolic cardiac function and calculated as follows: %FS = (LVIDd – LVIDs)/LVIDd * 100.

### Forelimb grip strength assay

Forelimb grip strength was measured as previously described^39^. Following acclimatization (at least one hour prior to grip strength measurement), each mouse was weighed and placed on a forelimb pull bar attached to an isometric force transducer (Columbus Instruments, Columbus, OH, USA). The mouse was pulled away from the bar by its tail, and the force required was recorded by the force transducer. Over 3 consecutive days, each mouse performed 3 pulls per day for a total of 9 pulls per test session. Peak tension force (N) was calculated as the average of each subject’s 9 pulls over the test session and normalized by body weight.

### Statistical analysis

All graphs and statistical comparisons were generated using GraphPad Prism 9. Statistical analyses were performed using the two-tailed unpaired *t*-test or one-way ANOVA followed by Tukey’s HSD test. All data are presented in this study as mean ± standard deviation (SD).

## Supplementary Information

Supplementary Information.

## Data Availability

The data that support the findings of this study are available from the corresponding author, RYW, upon reasonable request. WGS FASTA sequences were uploaded to the National Institutes of Health National Library of Medicine submission portal.

## References

[1] Fuller DD (2013). The respiratory neuromuscular system in Pompe disease. Respir. Physiol. Neurobiol..

[2] Slonim AE (2000). Identification of two subtypes of infantile acid maltase deficiency. J. Pediatr..

[3] Reuser, A., R. Hirschhorn, and M.A. Kroos, *Pompe disease: Glycogen storage disease type II, acid α-glucosidase (acid maltase) deficiency*, in *The Online Metabolic and Molecular Bases of Inherited Disease . Lysosomal Storage Disorders*, A. Beaudet, et al., Editors. 2018, The McGraw-Hill Companies, Inc..

[4] Hirschhorn R, Reuser AJJ, Scriver CR (2001). Glycogen storage disease type II: acid-glucosidase (acid maltase) deficiency. The metabolic and molecular basis of inherited disease.

[5] Kohler L, Puertollano R, Raben N (2018). Pompe disease: From basic science to therapy. Neurotherapeutics.

[6] Prater SN (2012). The emerging phenotype of long-term survivors with infantile Pompe disease. Genet. Med..

[7] Niño MY (2019). Extension of the Pompe mutation database by linking disease-associated variants to clinical severity. Hum. Mutat..

[8] Reuser AJJ (2019). GAA variants and phenotypes among 1079 patients with Pompe disease: Data from the Pompe Registry. Hum. Mutat..

[9] de Faria DOS (2021). Update of the Pompe variant database for the prediction of clinical phenotypes: Novel disease-associated variants, common sequence variants, and results from newborn screening. Hum. Mutat..

[10] Amarinthnukrowh P (2010). p.D645E of acid α-glucosidase is the most common mutation in Thai patients with infantile-onset Pompe disease. Genet. Test Mol. Biomark..

[11] Ko TM (1999). Molecular genetic study of Pompe disease in Chinese patients in Taiwan. Hum. Mutat..

[12] Shieh JJ, Lin CY (1998). Frequent mutation in Chinese patients with infantile type of GSD II in Taiwan: Evidence for a founder effect. Hum. Mutat..

[13] Doench JG (2016). Optimized sgRNA design to maximize activity and minimize off-target effects of CRISPR-Cas9. Nat. Biotechnol..

[14] O'Brien A, Bailey TL (2014). GT-Scan: Identifying unique genomic targets. Bioinformatics.

[15] Huang JY (2020). CRISPR-Cas9 generated Pompe knock-in murine model exhibits early-onset hypertrophic cardiomyopathy and skeletal muscle weakness. Sci. Rep..

[16] Behringer, R., et al., *Manipulating the Mouse Embryo: A Laboratory Manual*. Fourth Edition ed. 2014: Cold Spring Harbor Laboratory Press.

[17] Perry MN, Smith CL (2022). Murine allele and transgene symbols: Ensuring unique, concise, and informative nomenclature. Mamm. Genome.

[18] Raben N (1998). Targeted disruption of the acid alpha-glucosidase gene in mice causes an illness with critical features of both infantile and adult human glycogen storage disease type II. J. Biol. Chem..

[19] Dubowitz V, Sewry C, Oldfors A (2020). Histological and Histochemical Stains and Reactions. Muscle Biopsy-A Practical Approach.

[20] Raben N (2008). Suppression of autophagy in skeletal muscle uncovers the accumulation of ubiquitinated proteins and their potential role in muscle damage in Pompe disease. Hum. Mol. Genet..

[21] Fukuda T (2006). Autophagy and mistargeting of therapeutic enzyme in skeletal muscle in Pompe disease. Mol. Ther..

[22] Farah BL, Yen PM, Koeberl DD (2020). Links between autophagy and disorders of glycogen metabolism—Perspectives on pathogenesis and possible treatments. Mol. Genet. Metab..

[23] The Jackson Laboratory. *BODY WEIGHT INFORMATION FOR C57BL/6J (000664)*. [cited 2022 May 4, 2022]; Available from: https://www.jax.org/jax-mice-and-services/strain-data-sheet-pages/body-weight-chart-000664.

[24] Shieh JJ, Wang LY, Lin CY (1994). Point mutation in Pompe disease in Chinese. J. Inherit. Metab. Dis..

[25] Wan L (2008). Identification of eight novel mutations of the acid alpha-glucosidase gene causing the infantile or juvenile form of glycogen storage disease type II. J. Neurol..

[26] Bijvoet AG (1998). Generalized glycogen storage and cardiomegaly in a knockout mouse model of Pompe disease. Hum. Mol. Genet..

[27] Colella P (2020). Gene therapy with secreted acid alpha-glucosidase rescues Pompe disease in a novel mouse model with early-onset spinal cord and respiratory defects. EBioMedicine.

[28] van den Hout HM (2003). The natural course of infantile Pompe's disease: 20 original cases compared with 133 cases from the literature. Pediatrics.

[29] Kishnani PS (2006). A retrospective, multinational, multicenter study on the natural history of infantile-onset Pompe disease. J. Pediatr..

[30] Anzalone AV (2019). Search-and-replace genome editing without double-strand breaks or donor DNA. Nature.

[31] Brinkman EK (2014). Easy quantitative assessment of genome editing by sequence trace decomposition. Nucleic Acids Res..

[32] Brinkman EK (2018). Easy quantification of template-directed CRISPR/Cas9 editing. Nucleic Acids Res.

[33] Li H, Durbin R (2009). Fast and accurate short read alignment with Burrows–Wheeler transform. Bioinformatics.

[34] Weber J (2016). Sentieon DNA pipeline for variant detection—Software-only solution, over 20× faster than GATK 3.3 with identical results. PeerJ PrePrints.

[35] Li H (2009). The Sequence Alignment/Map format and SAMtools. Bioinformatics.

[36] Chen X (2016). Manta: Rapid detection of structural variants and indels for germline and cancer sequencing applications. Bioinformatics.

[37] Fujimoto A (1976). Two alpha-glucosidases in cultured amniotic fluid cells and their differentiation in the prenatal diagnosis of Pompe's disease. Clin Chim Acta.

[38] Schindelin J (2012). Fiji: An open-source platform for biological-image analysis. Nat Methods.

[39] Bonetto A, Andersson DC, Waning DL (2015). Assessment of muscle mass and strength in mice. Bonekey Rep.

