# Supplementary material for: CRISPR-mediated generation and characterization of a Gaa homozygous c.1935C>A (p.D645E) Pompe disease knock-in mouse model recapitulating human infantile onset-Pompe disease
Source: Sci Rep. 2022 Dec 14;12:21576. doi: 10.1038/s41598-022-25914-8 (PMC9751086; doi:10.1038/s41598-022-25914-8)
Supplement: Supplementary file 1 — Supplementary Information. [file 41598_2022_25914_MOESM1_ESM.docx]

**Supplementary Figures for**

**Title:**

CRISPR-Mediated Generation and Characterization of a *Gaa* Homozygous c.1935C>A (p.D645E) Pompe Disease Knock-in Mouse Model Recapitulates Human Infantile Onset-Pompe Disease

**Authors:**

Shih-hsin Kan^1#^, Jeffrey Y. Huang^1#^, Jerry Harb^1^, Allisandra Rha^1^, Nancy D. Dalton^1^, Chloe Christensen^1^ Yunghang Chan^2^, Jeremy Davis-Turak^3^, Jonathan Neumann^4^, Raymond Y. Wang^5,6*^

**Affiliation:**

^1^CHOC Children’s Research Institute, Orange, CA 92868

^2^School of Medicine, New York Medical College, NY 10595

^3^ROSALIND, San Diego, CA, 92126

^4^Transgenic Mouse Facility, University of California Irvine, Irvine, CA 92697

^5^Division of Metabolic Disorders, CHOC Children’s Specialists, Orange, CA 92868

^6^Department of Pediatrics, University of California-Irvine, Irvine, CA 92697

^#^ These authors contributed equally to this work

^*^Correspondence should be addressed to: RYW (rawang@choc.org)

**Supplementary Figure Legends:**

**Supplementary Figure 1: PAS and PAS-D staining showing glycogen storage in *Gaa^em1935C>A^* knock-in mouse tissues.** Top rows are the same images shown in Figure 4A in the main text. Bottom rows are adjacent slide sections of the same tissue showing PAS-D staining in muscle tissues. Abnormal glycogen accumulation in cardiac and skeletal muscle tissues was observed in KI mice compared to WT mice (top). Staining is no longer seen after diastase treatment, providing evidence for glycogen accumulation. Scale bar represents 100 µm.

**Supplementary Figure 2: Original unprocessed images for Figure 5.** Representative stain-free gel (left) and western blot (right) images from tissue homogenate from heart (A), diaphragm (B), gastrocnemius (GASTROC; C), and brain (D) of WT (n=3), HET (n=3), KI (n=4) and *Gaa^tm1Rabn^* (KO, n=3). LC3B-I and LC3B-II protein were probed against a primary LC3B antibody (highlighted in red boxes) and normalized to the amount of total protein as determined by densitometric analysis of stain-free gels.

**Supplementary Figure 3: No gender difference in** **anatomical features of left ventricular cardiac hypertrophy in 3-month-old** ***Gaa^em1935C>A^* knock-in mice.** Comparison of ventricular measurements and myocardial contraction in (A) male and (B) female mice. All measurements from WT (n=12; 6F, 6M), HET (n=10; 5F, 5M), and KI (*Gaa^.Em1935C>A^*; n=10; 5F, 5M) mice. Data are shown as mean ± SD. All comparisons were analyzed using one-way ANOVA with the Tukey post-hoc test. **p*<0.05, ***p*<0.01, ****p*<0.001, *****p*<0.0001, ns: not significant.

**Supplementary Table Legend:**

**Supplementary Table 1: *in silico* predicted off-target sites by GT-Scan.** Inputting gRNA-3 into GT-Scan yields four predicted off-target sites utilizing the conditions “SpCas9,” standard ruleset “xxxxxxxxxxXXXXXXXXXXNGG,” standard off-target filter “NNNNNNNNNNNNNNNNNNNNNRG,” and 3 mismatches. GT-Scan yields three predicted off-target sites for gRNA-2 utilizing the same conditions. Mismatched nucleotides are lowercase. PAM sequences in green.

**Supplementary Figure 1.**

**
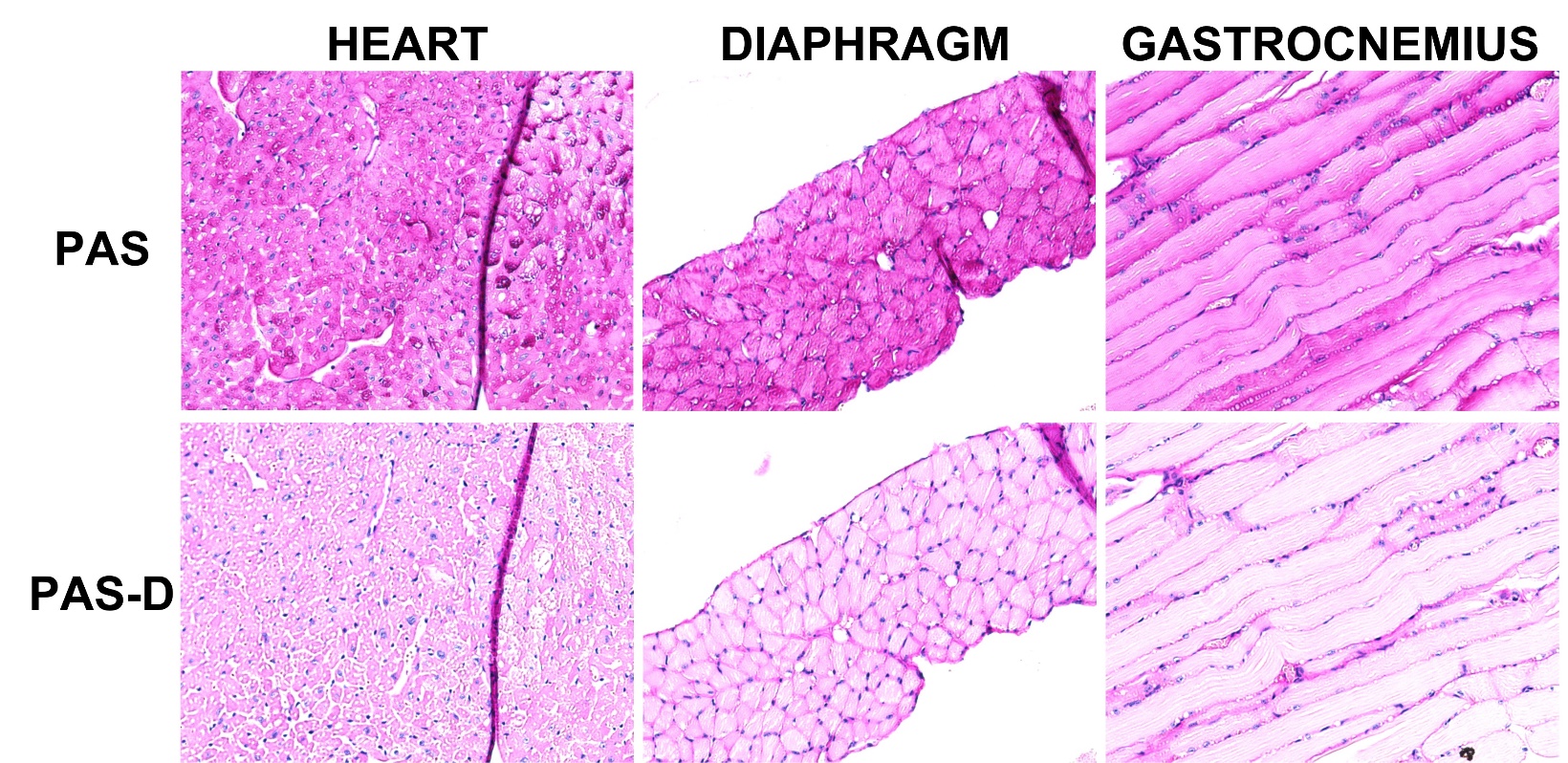
**

**Supplementary Figure 2.**


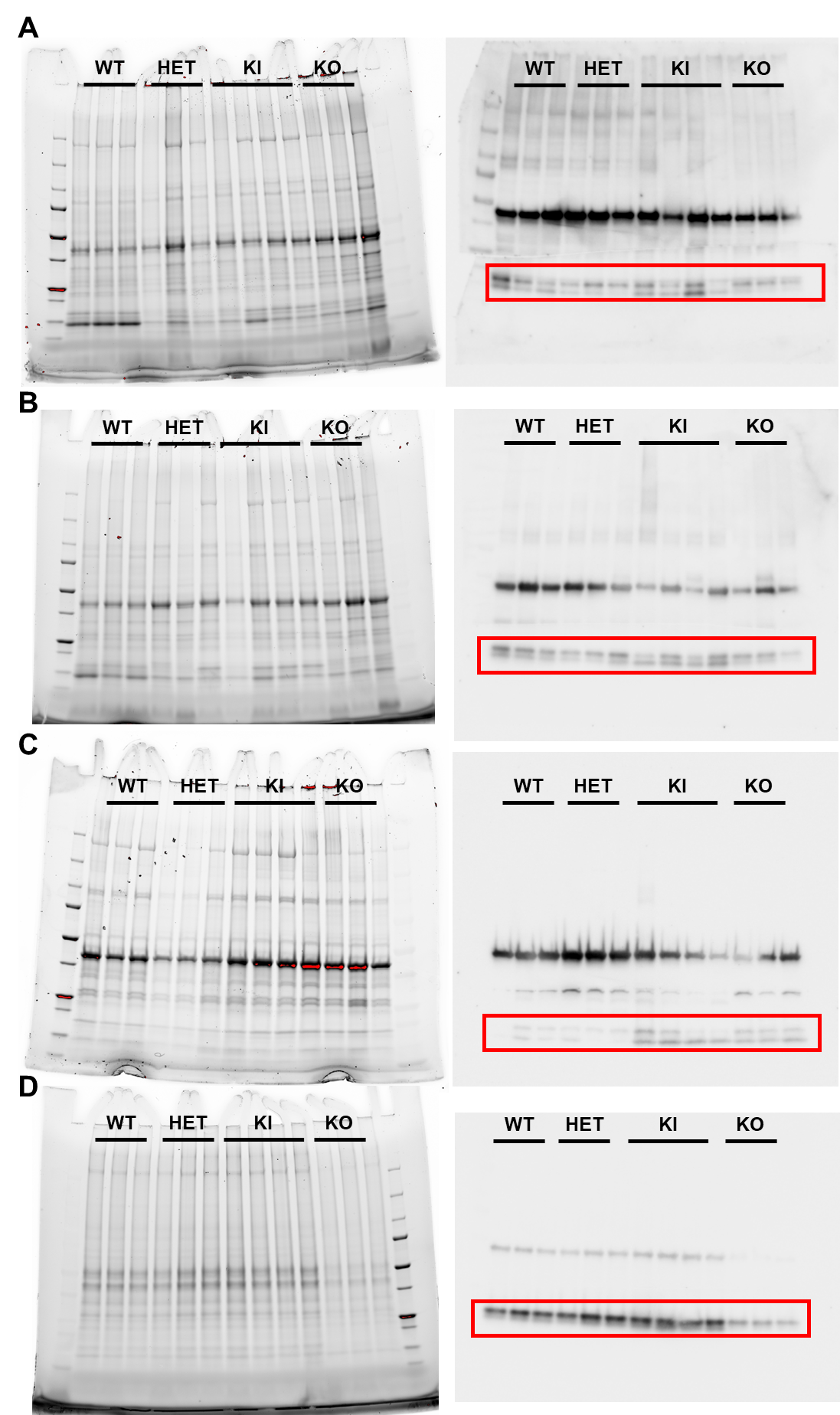


**Supplementary Figure 3.**

**
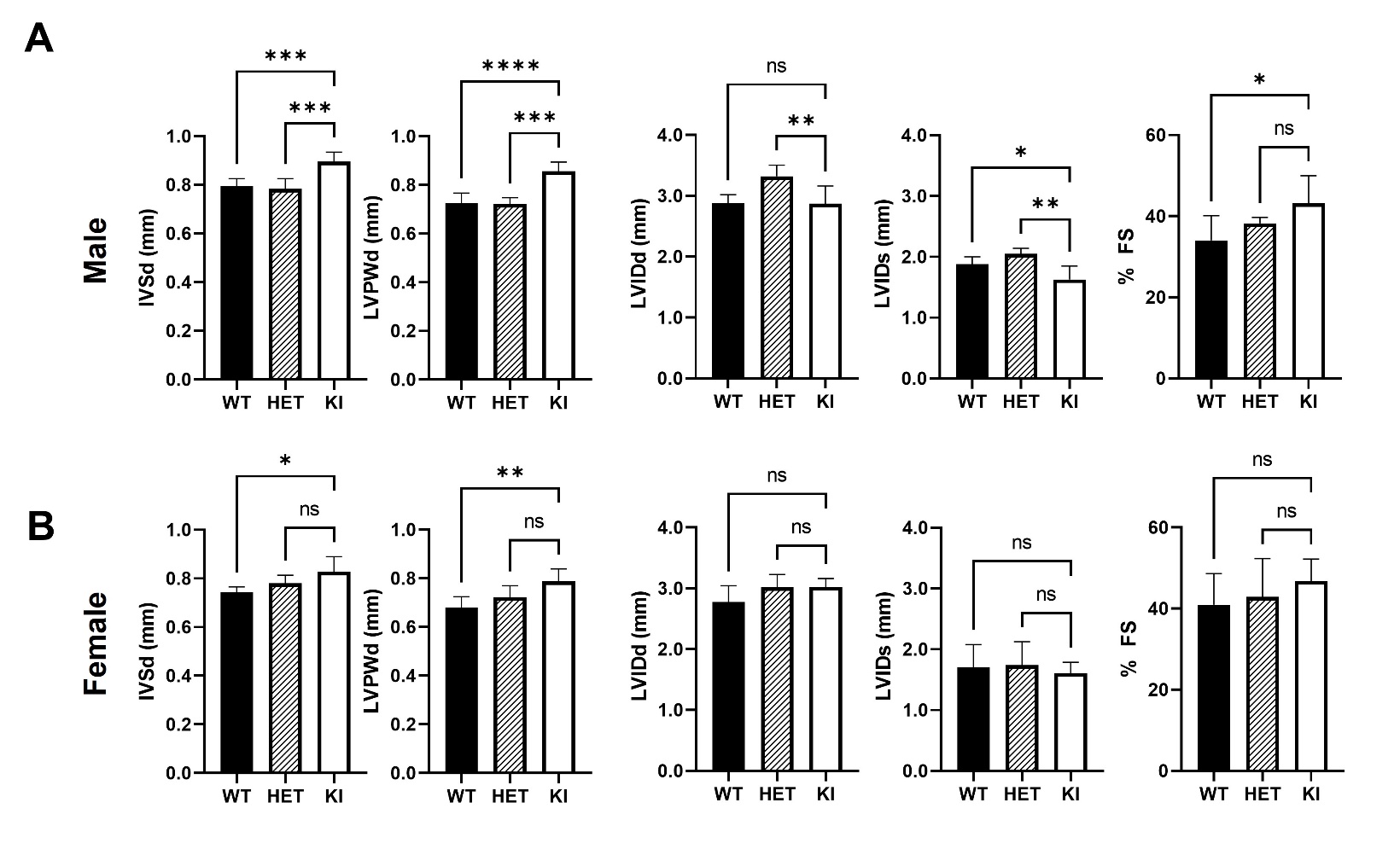
**

**Supplementary Table 1.**

GGGCGTGCCCCTGGTCGGGGCGG [Chromosome 11,Position 119280434, *Gaa* gene gRNA-3]

OT1) GGGaGTGtCCCTGGTCGGGGCaG [Chromosome 2, Position 33211326, intronic]

OT2) GGGCtTcCCCCTGGTCtGGGGGG [Chromosome 9, Position 64173456, *Rpl4* promoter]

OT3) GGGCGTGgCCCTGGgaGGGGCGG [Chromosome 7, Position 30281088, intronic]

OT4) GGGCGTGgCCCTGGTgGGGatGG [Chromosome X, Position 70701496, intronic]

GCAGATGTCCGCCCCGACCAGGG [Chromosome 11, Position 119280445, *Gaa* gene gRNA-2]

OT5) GCAcgaGTCCGCCCCGACCAGGG [Chromosome 3, Position 26331520, *Nlgn1* gene]

OT6) GaAGATGTCCGCCCaGACCaaGG [Chromosome 16, Position 30671855, intronic]

OT7) GCAGgTGTCCGCCCCGgCCtGGG [Chromosome 18, Position 7028979, intronic]
